# CD248/endosialin critically regulates hepatic stellate cell proliferation during chronic liver injury via a PDGF-regulated mechanism

**DOI:** 10.1136/gutjnl-2014-308325

**Published:** 2015-06-15

**Authors:** Annika Wilhelm, Victoria Aldridge, Debashis Haldar, Amy J Naylor, Christopher J Weston, Ditte Hedegaard, Abhilok Garg, Janine Fear, Gary M Reynolds, Adam P Croft, Neil C Henderson, Christopher D Buckley, Philip N Newsome

**Affiliations:** 1 NIHR Birmingham Liver BRU and Centre for Liver Research, University of Birmingham, Birmingham, UK; 2 University Hospital Birmingham NHS Foundation Trust, Birmingham, UK; 3 Centre for Translational Inflammation Research, University of Birmingham, Birmingham, West Midlands, UK; 4 MRC Centre for Inflammation Research, University of Edinburgh, Edinburgh, UK

**Keywords:** HEPATIC STELLATE CELL, MYOFIBROBLASTS, FIBROSIS

## Abstract

**Introduction:**

CD248 (endosialin) is a stromal cell marker expressed on fibroblasts and pericytes. During liver injury, myofibroblasts are the main source of fibrotic matrix.

**Objective:**

To determine the role of CD248 in the development of liver fibrosis in the rodent and human setting.

**Design:**

CD248 expression was studied by immunostaining and quantitative PCR in both normal and diseased human and murine liver tissue and isolated hepatic stellate cells (HSCs). Hepatic fibrosis was induced in CD248^−/−^ and wild-type controls with carbon tetrachloride (CCl_4_) treatment.

**Results:**

Expression of CD248 was seen in normal liver of humans and mice but was significantly increased in liver injury using both immunostaining and gene expression assays. CD248 was co-expressed with a range of fibroblast/HSC markers including desmin, vimentin and α-smooth muscle actin (α-SMA) in murine and human liver sections. CD248 expression was restricted to isolated primary murine and human HSC. Collagen deposition and α-SMA expression, but not inflammation and neoangiogenesis, was reduced in CD248^−/−^ mice compared with wild-type mice after CCl_4_ treatment. Isolated HSC from wild-type and CD248^−/−^ mice expressed platelet-derived growth factor receptor α (PDGFR-α) and PDGFR-β at similar levels. As expected, PDGF-BB stimulation induced proliferation of wild-type HSC, whereas CD248^−/−^ HSC did not demonstrate a proliferative response to PDGF-BB. Abrogated PDGF signalling in CD248^−/−^ HSC was confirmed by significantly reduced c-fos expression in CD248^−/−^ HSC compared with wild-type HSC.

**Conclusions:**

Our data show that deletion of CD248 reduces susceptibility to liver fibrosis via an effect on PDGF signalling, making it an attractive clinical target for the treatment of liver injury.

**Significance of this study**

**What is already known on this subject?**

- Fibrosis key is a challenge in hepatology for which new therapies are needed.
- Activation of hepatic stellate cells is critical to the development of liver fibrosis.

**What are the new findings?**

- CD248 is upregulated at gene and protein level in both murine and human liver fibrosis.
- Deletion of CD248 reduces development of liver fibrosis during chronic liver injury.
- CD248 effect is mediated through abrogated platelet-derived growth factor signalling in hepatic stellate cells.

**How might it impact on clinical practice in the foreseeable future?**

- CD248 is an attractive target for antifibrotic drugs and merits consideration in clinical trials.

## Introduction

Progressive liver fibrosis represents one of the major consequences of chronic hepatic injury and is associated with the activation of hepatic stellate cells (HSCs) from a quiescent state into proliferative, α-smooth muscle actin (α-SMA)-positive myofibroblasts that produce a variety of extracellular matrix (ECM) proteins.^1^ Identification of mechanisms central to the development of liver fibrosis offers the potential to target these molecules in the development of antifibrotic therapies.

CD248, also known as endosialin and tumour endothelial marker 1, is a trans-membrane glycoprotein initially believed to be expressed on endothelial cells of different cancers including colon^2^ and brain.^3^ Its expression was subsequently shown to be on perivascular NG2+ cells (pericytes) in the tumour vasculature as well as on interstitial fibroblasts^4^ and mesenchymal stem cells.^5^ Outside the setting of cancer, CD248 appears to be developmentally regulated with expression occurring early during embryonic development,^6^ although it is not essential for development as CD248-deficient mice are viable and fertile.^7^

CD248 expression largely disappears in the adult, only reappearing during infection, tissue repair^6^ and cancer.^5^ In the setting of cancer, CD248 expressing stromal cells interact with potential ligands including fibronectin and collagen types I and IV and exhibit enhanced adhesion and migration through Matrigel supporting a role for CD248 in tumour progression and invasion.^8^ A role for CD248 has been confirmed in the setting of chronic kidney disease, where CD248 is expressed on myofibroblasts and has been demonstrated to serve as marker for both the extent of renal fibrosis and disease outcome.^9^

The role of CD248 in liver fibrosis has not yet been established, but given its potential role in other fibrotic diseases, we sought to evaluate the function of CD248 in chronic liver injury. Herein, we demonstrate that CD248 expression is upregulated in hepatic fibrosis where its expression is confined to HSCs and myofibroblasts. Moreover, we illustrate that genetic deletion of CD248 protects against the development of fibrosis, despite similar levels of liver injury, inflammation and neoangiogenesis, in a murine model of chronic hepatocellular injury by interfering with platelet-derived growth factor (PDGF) signalling, and therefore represents a novel target for therapeutic intervention in fibrotic liver injury.

## Materials and methods

### Human liver tissue

Tissue was obtained with ethical approval and consent from the liver unit at Queen Elizabeth Hospital Birmingham, UK. Explanted human liver specimens were obtained from patients with alcoholic liver disease, non-alcoholic steatohepatitis, primary biliary cirrhosis and autoimmune hepatitis. Normal donor liver surplus to surgical requirements or as a by-product of surgical resection served as normal controls.

### Mice

C57Bl/6 CD248^−/−^7 mice were a gift from David Huso (Johns Hopkins University, USA). Wild-type C57Bl/6 (CD248^+/+^, hereafter termed WT) were purchased from Harlan Laboratories, UK. Six-week-old to 10-week-old CD248^−/−^ and WT mice received twice-weekly intraperitoneal carbon tetrachloride (CCl_4_) injections (0.25 mL/kg (Sigma)) for 8 or 12 weeks to induce chronic liver injury and were sacrificed 2 days after the last dose. Mineral oil was used as vehicle. In some instances, mice received CCl_4_ for 8 weeks and were sacrificed 4 weeks after administration of last dose (resubmission group). All animal procedures were conducted in accordance with UK laws with the approval of the Home Office and local ethics committees (PPL 40/3201). To assess liver function, alanine aminotransferase (ALT) activity was measured in serum samples at the clinical biochemistry laboratory of the Women's Hospital Birmingham (Birmingham, UK).

### Isolation of primary human and murine liver-derived cells

Primary human and mouse HSCs were isolated and grown as described previously.^10^ ^11^ Human HSCs were isolated from uninvolved liver tissue of patients undergoing surgical resection for primary or secondary liver malignancy. Murine HSCs were isolated from normal livers. HSCs were isolated by pronase/collagenase digestion and separated by buoyancy centrifugation. HSC activated spontaneously in culture over 10 days with loss of retinoic acid, phenotypic changes and up-regulation of α-SMA. HSC cultures were in some instances stimulated with PDGF-BB (1–100 ng/mL; Peprotech, London, UK) for 24 h. Isolation of biliary epithelial cells, endothelial cell, hepatocytes and liver myofibroblasts was performed using explanted end-stage diseased liver tissue as described previously.^10^ ^12^

### Light microscopy and immunohistochemistry

Paraffin-embedded mouse liver tissue cut to 3 μm thickness was stained with H&E using standard protocols. For immunohistochemical studies, paraffin-embedded mouse liver sections were incubated with optimally diluted rabbit antimouse vimentin, rabbit antimouse desmin, rabbit antimouse F4/80 (all Abcam, Cambridge, UK) or rat antimouse CD45 (eBioscience, Hatfield, UK) using a Dako Autostainer. Staining was detected with immPRESS antirabbit secondary antibody and visualised in NovaRED chromagen (both Vector Laboratories, Peterborough, UK). Sections were then counterstained with haematoxylin (VWR International, Leighton Buzzard, UK). For CD45+ immune cell quantification, five non-overlapping fields from each stained section were captured using a light microscope (Zeiss, Germany) with identical illumination and exposure. Digital image analysis was performed using ImageJ software. CD45 values were expressed as the percentage of the total area of the section occupied by CD45 staining.

### Immunofluorescent staining

Liver tissue was snap-frozen and cut into 7-μm-thick sections on a cryostat. The sections were fixed for 5 min in acetone. Immunofluorescent staining on cells was carried out with 100% methanol fixed primary cells grown on glass coverslips. For human sections and primary human HSCs, the following antibodies were used: monoclonal mouse IgG1 antihuman CD248 (generated in-house), monoclonal mouse IgG2a antihuman α-SMA (Dako, Ely, UK), monoclonal mouse IgG1 antihuman glial fibrillary acidic protein (GFAP) (Dako), monoclonal mouse IgG1 antihuman vimentin (Vector Laboratories) and monoclonal mouse IgG1 antihuman CD90 (eBioscience). Murine sections and primary mouse HSCs were stained with the following antibodies: polyclonal rabbit antimouse CD248 (generated in-house by Prof. Clare Isacke, Institute of Cancer Research), polyclonal rabbit antimouse α-SMA, polyclonal goat antimouse GFAP, polyclonal rabbit antimouse desmin (all from Abcam), monoclonal rat IgG2a antimouse PDGFR-α and monoclonal rat IgG2a antimouse PDGFR-β (both from eBioscience) hamster antimouse CD31 (AbD Serotec, BioRad, USA). In instances when antibodies had the same isotype, sequential staining with separate antibody incubations was carried. Species isotype (Life Technologies, Paisley, UK) staining controls were routinely performed. The primary antibodies were detected with fluorescently conjugated secondary antibodies and in some cases tertiary antibodies followed by DAPI as a nuclear counterstain. Sections and cells were mounted in fluorescent mountant (Dako). The slides were visualised using a Zeiss confocal LSM 510 microscope (Zeiss) and processed using Zeiss LSM Image Examiner software (Zeiss). Representative images are shown. For quantitative analysis, stained sections were scanned in an automated fashion using identical settings on an AxioScan Z1 (Zeiss) slide scanner and arithmetic mean pixel counts were analysed using Zen Lite 2012 (Zeiss).

### Picrosirius red staining and quantification

Sections were hydrated with distilled water, then placed into 0.5% phosphomolybdic for 5 min, before staining with 0.1% Sirius red (direct red 80; % w/v in saturated picric acid) for 2 h. The slides were dipped in 0.01 M HCl, before washing in distilled water and then mounted. To quantify Picrosirius red staining (PSR), images of five or six random fields of each section were taken and processed with ImageJ software. Collagen values were expressed as the percentage of the total area of the section occupied by Sirius red staining.

### Proliferation assay

Ki-67 staining was conducted for quantitative evaluation of the HSC proliferation rate using the polyclonal rabbit anti-Ki-67 antibody (Millipore, USA). The number of Ki-67-positive nuclei was counted and expressed as the percentage of the total number of nuclei to determine the frequency of Ki-67-positive HSC.

### qPCR analysis

Total RNA was extracted by Qiagen RNeasy Mini Kit (Qiagen, California, USA) according to manufacturer's instructions. Complementary DNA (cDNA) was synthesised from 1 μg of RNA using iScript cDNA Synthesis Kit (Biorad, UK). The cDNA was then amplified by qPCR with a Lightcycler 480 using probes (Roche, UK) and primers (Alta Biosciences, UK; see table 1). For further mouse studies, TaqMan gene expression assays (Life Technologies, UK) were used; CD248 (Mm00547485_s1), Tnf-α (Mm00443258_m1), Il6 (Mm00446190_m1), Il10 (Mm00439614_m1), Pdgfb (Mm00440677_m1), Pdgfra (Mm00440701_m1), Pdgfrb (Mm00435546_m1), Tgfb1 (Mm01178820_m1), Ctgf (Mm01192932_g1), Hgf (Mm01135193_m1), Timp1 (Mm00441818_m1), Mmp2 (Mm00439498_m1) and Mmp9 (Mm00442991_m1). Expression levels of the target genes were normalised to a housekeeping gene *Gapdh* (Mm99999915_g1). Gene expression values are stated as 2^−ΔCt^.

**Table 1:** Primers used for qPCR analysis

Target	Forward primer	Reverse primer
Human CD248	ACCTCGGAGATGAGTTGCTG	TTCCAGGCCTCGTCTTCAT
Mouse α-SMA	CCGCCATGTATGTGGCTATT	CAGTTGTACGTCCAGAGGCATA
Mouse Col1a1	AGACATGTTCAGCTTTGTGGAC	GCAGCTGACTTCAGGGATG
Mouse c-fos	CAGCCTTTCCTACTACCATTCC	ACAGATCTGCGCAAAAGTCC

α-SMA, α-smooth muscle actin.

### Western blots

Cell lysates were prepared by the addition of sodium dodecyl sulfate (SDS) sample buffer (4% SDS (v/v), 0.1 M dithiothreitol, 20% glycerol (v/v), 0.0625 M Tris–HCl and 0.004% bromophenol blue (w/v) and heated for 10 min at 100°C. Protein was resolved on a 4–12% Novex Tris-glycine gel (Invitrogen Life Technologies, Paisley, UK) and transferred to Hybond-N membranes (GE Healthcare Life Sciences, Buckinghamshire, UK) using a Novex X-Cell II Mini Cell. Blots were reacted with rabbit antimouse total or phospho-p44/42 MAPK (Erk1/2) (Thr202/Tyr204) (197G2, New England Biolabs, Herts, UK). Detection was subsequently performed with species-specific secondary antibodies conjugated to horseradish peroxidase (GE Healthcare Life Sciences). Protein loading was confirmed by antimouse β-actin antibody (eBioscience). Blots were visualised by enhanced chemiluminescence (GE Healthcare Life Sciences) and digital images and densitometry performed using a chemi-doc Bio-Rad system. Data were expressed as relative quantification normalised to β-actin expression and presented as fold change from unstimulated cells.

### Statistical analysis

Statistical analysis was performed by Student's t test or Spearman's correlation coefficient using Prism software (GraphPad, California, USA). Data are expressed as mean with SEs. A value of p<0.05 was considered significant.

## Results

### Expression of hepatic CD248 increases at both the gene and protein level during liver injury in mouse and human and correlates with levels of fibrosis

**Figure 1:**
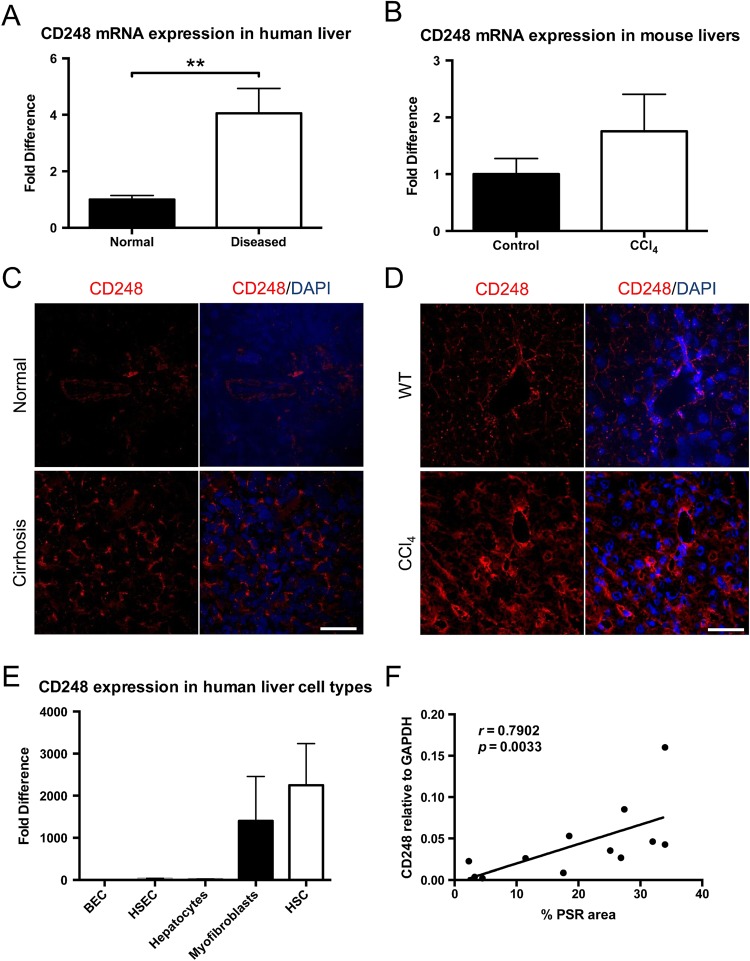
CD248 expression is upregulated in chronic liver disease and correlates with levels of fibrosis. CD248 mRNA was analysed by qPCR (A) from normal human livers (n=12) versus end-stage diseased cirrhotic livers (n=18) and (B) livers from untreated mice (control) and mice injected with carbon tetrachloride (CCl_4_) twice weekly for 8 weeks (n=6). Data represent CD248 mRNA fold changes and are shown as mean±SEM. **p<0.01 (Student's t test). Representative images of CD248 immunofluorescent staining (red) (C) in normal and cirrhotic human liver tissue and (D) in livers from untreated (wild-type) and CCl_4_ injected mice. Scale bar=50 μm. (E) RNA was isolated from primary biliary epithelial cells (BEC), hepatic sinusoidal endothelial cells (HSEC), hepatocytes, myofibroblasts and activated hepatic stellate cells (n=3 different isolates) and analysed by qPCR for expression levels of CD248 mRNA. Results are expressed as mean±SD. (F) Normal and cirrhotic livers (n=12) were stained using Sirius red. Staining was digitally quantified and expressed as the percentage area covered by staining of five randomly selected fields per sample. Extent of fibrosis was compared with CD248 mRNA (r=0.79; p<0.01; Spearman's r correlation). PSR, Picrosirius red staining.

Expression levels of CD248 mRNA were higher in human cirrhotic end-stage liver disease (3.85-fold difference; p<0.01) as compared with normal human liver (figure 1A). A similar pattern was observed in chronic CCl_4_-induced liver fibrosis in mouse liver where CD248 expression also appeared higher (1.6-fold difference; p=0.09) than in uninjured mouse liver (figure 1B). Immunofluorescent staining for CD248 revealed occasional positive cells in normal human liver with a marked increase in the number of CD248^+^ cells seen in cirrhotic livers (figure 1C). CD248 was also expressed at low levels in healthy mouse livers, whereas a considerably higher number of CD248^+^ cells were observed in chronically injured mouse livers (figure 1D). Having established that CD248 expression increased during liver injury, we sought to determine which cells in the liver were expressing CD248. Isolated HSCs from normal livers that were activated on plastic and myofibroblasts from human cirrhotic livers expressed high levels of CD248 mRNA in contrast to human hepatic sinusoidal endothelial cells, biliary epithelial cells and hepatocytes isolated from cirrhotic livers (figure 1E). In addition, a positive correlation was detected between CD248 mRNA and the amount of collagen in human livers (Spearman's r=0.79; p<0.01; figure 1F).

### CD248 is expressed on myofibroblast-like cells in sinusoids and around blood vessels in both human and mouse tissue

**Figure 2:**
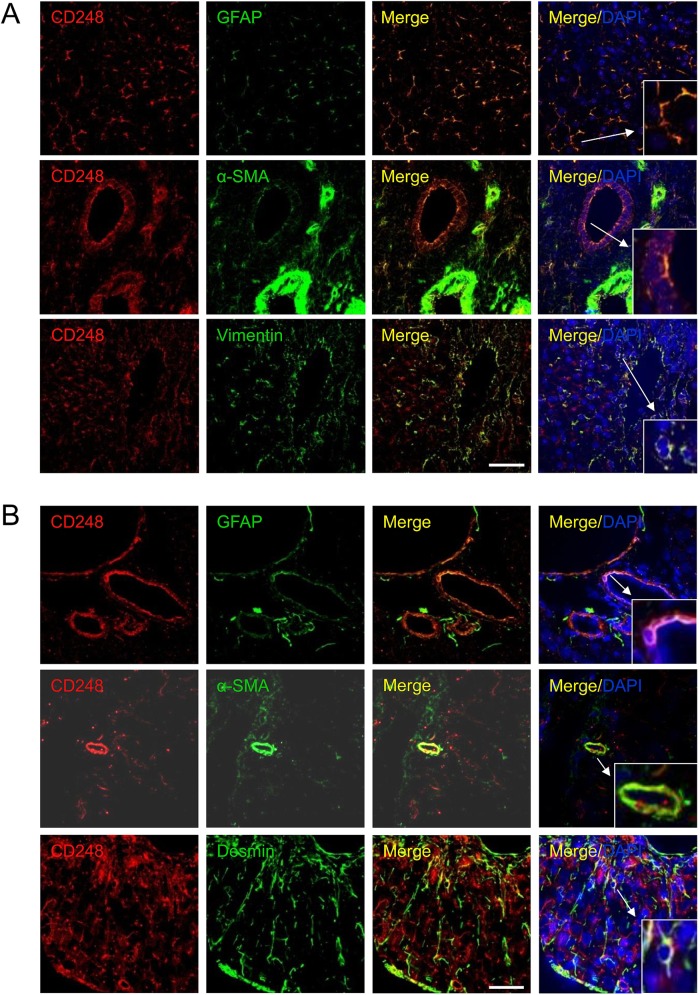
CD248 is expressed by stromal cells in the human and mouse liver. Representative confocal images showing localisation of CD248 (red) to cells expressing myofibroblast and hepatic stellate cell markers (green) located in blood vessels and sinusoids in (A) human chronic end-stage liver and (B) chronically injured mouse liver sections. DAPI was used as a counterstain. Insets; zoomed images. Scale bar=50 μm. GFAP, glial fibrillary acidic protein; α-SMA, α-smooth muscle actin.

**Figure 3:**
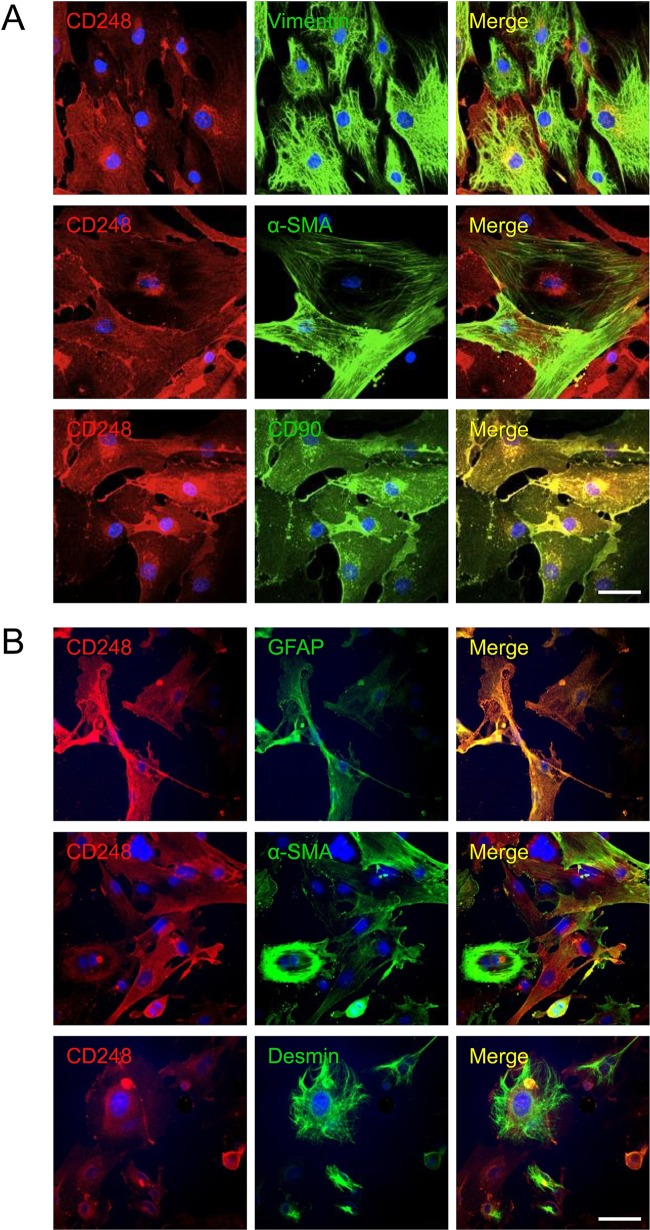
CD248 expression in stromal cells in vitro from human and mouse liver. Hepatic stellate cells (HSCs) were isolated from normal (A) human and (B) mouse livers and activated on plastic. Representative confocal images showing CD248 expression (red) in human and mouse HSC expressing HSC markers. DAPI was used as a counterstain. Scale bar=50 μm. GFAP, glial fibrillary acidic protein; α-SMA, α-smooth muscle actin.

To further investigate the expression and localisation of CD248 in injured livers, we performed immunofluorescent dual staining. CD248 expression was located in the sinusoids and around blood vessels of cirrhotic human liver and co-localised with cells expressing a range of myofibroblast/HSC markers including GFAP, α-SMA and vimentin (figure 2A). The distribution of CD248^+^ cells was similar in CCl_4_-induced liver fibrosis, again being localised to the sinusoids and blood vessels. Moreover, CD248^+^ cells in injured murine liver co-localised with a range of relevant murine fibroblast markers including GFAP, α-SMA and desmin (figure 2B). To confirm the identity of the cells, we isolated quiescent human and murine HSC from normal livers that then activated in culture and co-stained them with CD248 and HSC markers. Activated HSCs isolated from human livers exhibited bright CD248 staining (figure 3A) as did mouse HSCs (figure 3B). Both human and murine HSCs co-expressed CD248 with other established stellate cell markers (figure 3).

**Figure 4:**
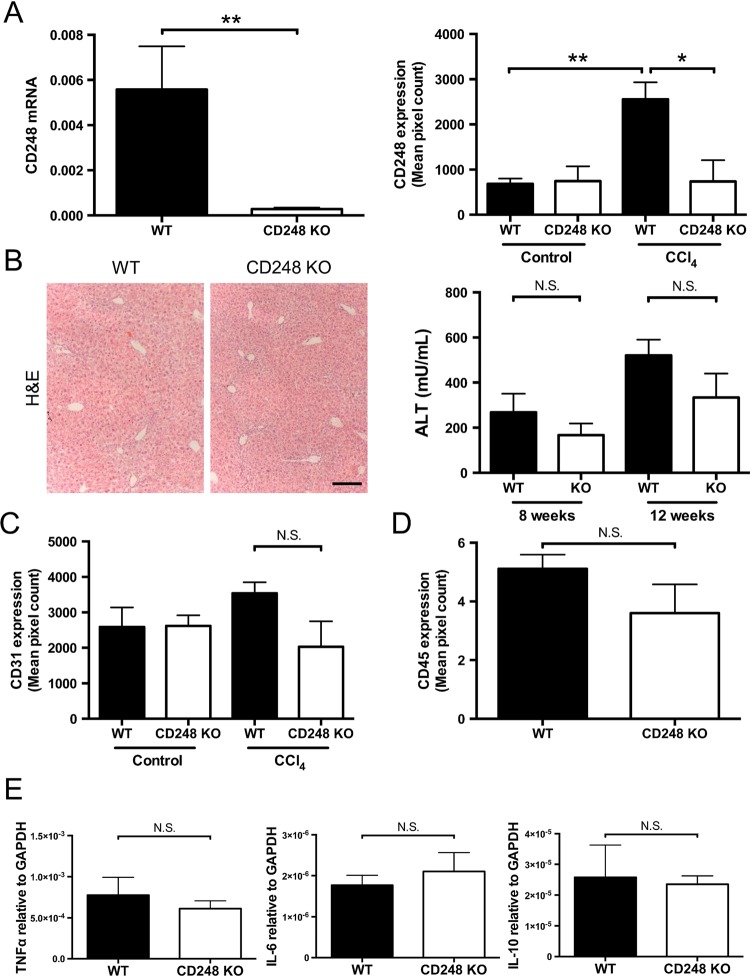
CD248 knock-out does not alter inflammatory response to carbon tetrachloride (CCl_4_) injury. CD248^−/−^ mice and wild-type (WT) mice were injected biweekly with CCl_4_ or vehicle for 8 weeks and then sacrificed after 2 days of the final dose (n=6). (A) Levels of CD248 mRNA and protein were quantified using qPCR and morphometric analysis. (B) Representative images of H&E staining of paraffin-embedded liver tissue sections are shown and serum alanine aminotransferase (ALT) activity was assessed as a measure of acute liver injury. Scale bar=200 μm. (C) Digital quantification of CD31 expressed as the percentage area per section. (D) Digital quantification of CD45 expressed as the percentage area of five randomly selected areas per sample. (E) Expression levels of inflammatory mediators were analysed by qPCR. Data are mean±SEM. *p<0.05, **p<0.01 (Student's t test). GAPDH, glyceraldehyde 3-phosphate dehydrogenase; TNF-α, tumour necrosis factor-α.

Having established that CD248 expression is restricted to HSCs and myofibroblasts, we sought to determine the functional significance of CD248 in the generation of liver fibrosis using a CD248-deficient murine model. After intraperitoneal injection of CCl_4_ for 8 weeks, we verified that expression of CD248 mRNA and protein were not increased in the liver (figure 4A). CD248^−/−^ mice had very low CD248 mRNA levels compared with their WT controls (p<0.01). In addition, minimal CD248 protein was detected in CD248^−/−^ mice following CCl_4_ injury compared with WT controls (p<0.05). In contrast, a significant increase of CD248 protein expression was detected in WT mice treated with CCl_4_ compared with vehicle only (p<0.01). Following CCl_4_ administration, CD248^−/−^ mice and WT mice both had foci of centrilobular necrosis as shown by H&E staining (figure 4B).

### CD248-deficient mice have similar levels of angiogenesis and inflammation following chronic CCl_4_-induced liver injury

Serum ALT activity was not different in CD248^−/−^ mice compared with WT mice after either 8 or 12 weeks of CCl_4_ administration (figure 4B). Also, levels of inflammation as assessed by hepatic CD45 protein expression (see figure 4D and online supplementary figure S1) and tumour necrosis factor-α, interleukin (IL)-6 and IL-10 mRNA (figure 4E) did not vary between CD248^−/−^ mice and WT mice. (figure 4B). There were no differences seen in expression of hepatic macrophages between the two groups (see online supplementary figure S1). Furthermore, no significant difference was detected in levels of angiogenesis as assessed by CD31 expression in CD248^−/−^ mice and WT mice treated with CCl_4_ for 8 weeks (figure 4C).

### CD248-deficient mice are protected from CCl_4_-induced hepatic fibrosis

**Figure 5:**
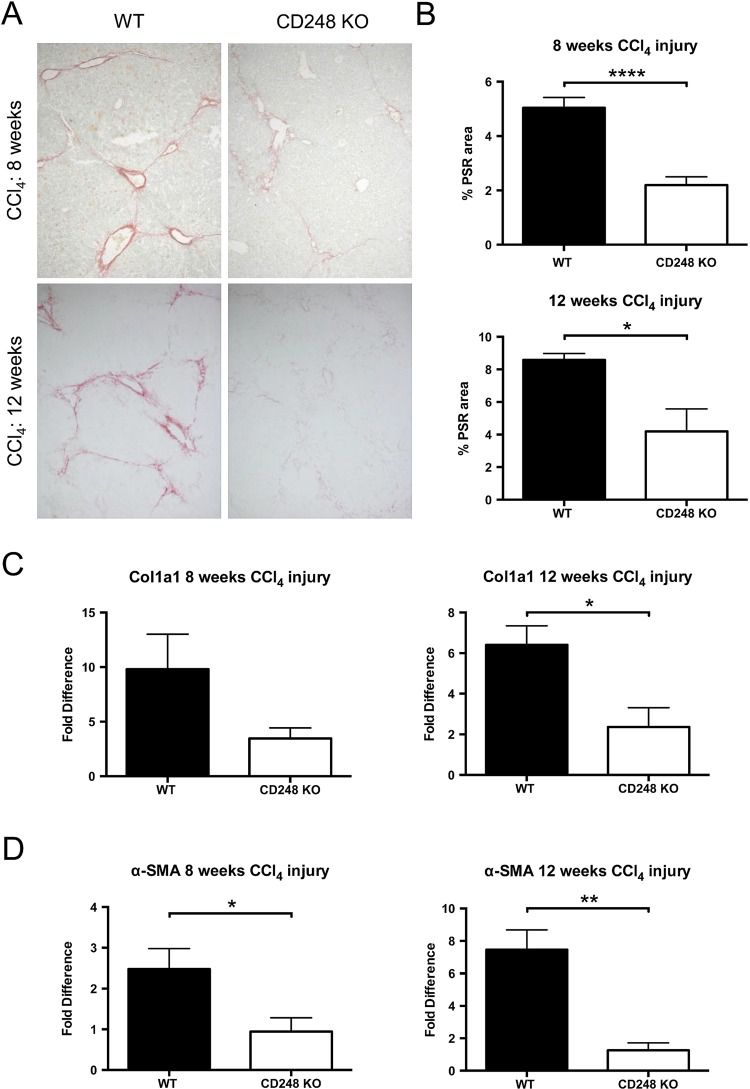
CD248-deficicient mice are protected from carbon tetrachloride (CCl_4_)-induced hepatic fibrosis. CD248^−/−^ mice and wild-type (WT) mice were injected biweekly with CCl_4_ for 8 or 12 weeks (n=6). (A) Liver sections were stained with Picrosirius red staining (PSR) to reveal collagen deposition. (B) The percentage fibrotic areas were assessed by ImageJ analysis of microphotographs taken of six randomly selected areas. (C) Col1a1 and (D) α-smooth muscle actin (α-SMA) gene expression analysis was performed with qPCR using total liver mRNA. Data are mean±SEM. *p<0.05, **p<0.01, ****p<0.0001 (Student's t test).

To further investigate the role of CD248 in liver fibrogenesis, we examined the extent of collagen deposition and myofibroblasts accumulation in CD248^−/−^ and WT mice. Whereas PSR in chronically injured WT mice revealed a significant accumulation of collagen in the portal area stretching into the lobules in livers after 8 and 12 weeks, collagen staining in CD248^−/−^ mice was scarcely observed (figure 5A). Digital morphometric analysis of PSR sections demonstrated a marked reduction in liver fibrosis in CD248^−/−^ mice compared with WT mice at both 8 (56.4% reduction; p<0.0001) and 12 weeks (51.1% reduction; p<0.05), respectively (figure 5B). To further quantify differences in fibrosis, levels of procollagen α 1(I), a component of collagen 1, and α-SMA mRNA were measured by qPCR (figure 5C, D). Procollagen α 1(I) expression was 65.3% and 64.0% less in CD248^−/−^ mice after 8 and 12 weeks (p<0.05) of CCl_4_ injury, respectively (figure 5C). Similarly, α-SMA expression (figure 5D) was reduced in CD248^−/−^ mice by 62.5% and 86.1% at 8 and 12 weeks, respectively (both p<0.05).

### The key fibrogenic factor TGF-β is reduced in CD248-deficient mice following CCl_4_-induced injury

**Figure 6:**
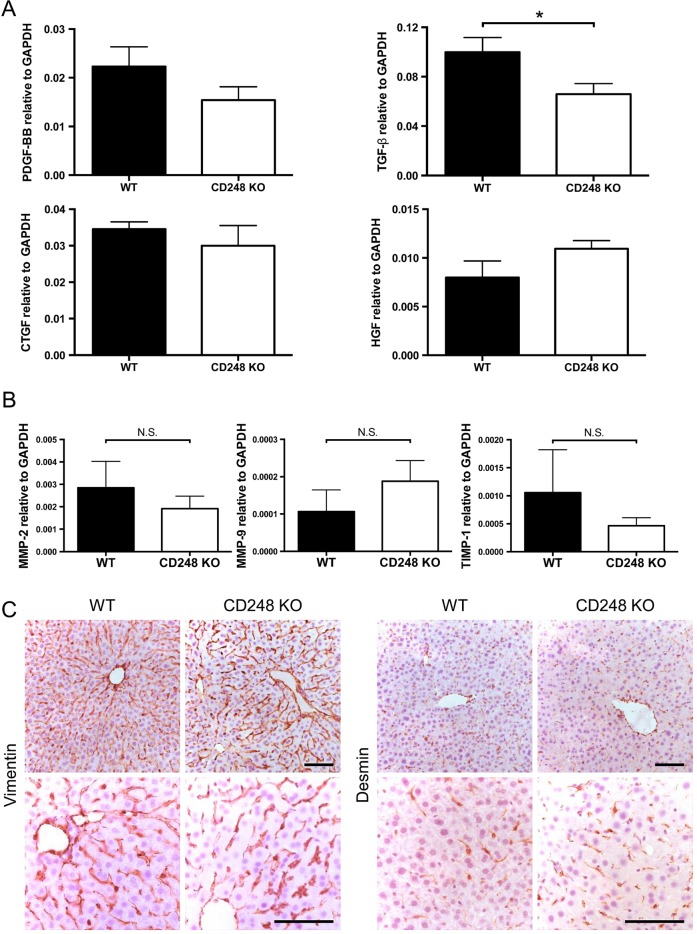
Hepatic transforming growth factor-β (TGF-β) is reduced in CD248-deficient mice following carbon tetrachloride (CCl_4_)-induced injury. CD248^−/−^ mice and wild-type (WT) mice were injected biweekly with CCl_4_ for 8 weeks (n=6). (A) Transcript levels of growth factors and (B) enzymes involved in matrix remodelling were analysed by qPCR of whole liver tissue. (C) Representative images of livers from untreated CD248^−/−^ mice and WT mice stained with vimentin or desmin are shown. Scale bar=100 μm. CTGF, connective tissue growth factor; GAPDH, glyceraldehyde 3-phosphate dehydrogenase; HGF, hepatocyte growth factor; MMP, matrix metalloproteinase; PDGF, platelet-derived growth factor; TIMP, tissue inhibitor of metalloproteinase.

To further dissect the role of CD248 in fibrosis, growth factors involved in fibrogenesis and liver injury were assessed following CCl_4_ administration. Expression levels of transforming growth factor-β (TGF-β) mRNA, which is a key pro-fibrogenic growth factor that regulates HSCs, were reduced in CD248^−/−^ mice compared with their WT controls (p<0.05), whereas the levels of PDGF-BB, connective tissue growth factor and hepatocyte growth factor mRNA did not differ (figure 6A). In addition, mRNA expression levels of enzymes involved in matrix remodelling including matrix metalloproteinase (MMP)-2, MMP-9 and tissue inhibitor of metalloproteinase-1 remained unchanged between CD248^−/−^ mice and WT mice following CCl_4_ administration (figure 6B). In order to exclude the possibility that deficiency of CD248 might have affected the abundance of HSCs, we performed histological examination between WT and CD248^−/−^ mice without CCl_4_-induced liver injury. Livers stained with two common markers for HSC, vimentin and desmin, demonstrated that the number of HSCs was not affected by genetic deletion of CD248 (figure 6C).

### Deficiency of CD248 does not affect resolution of liver fibrosis

**Figure 7:**
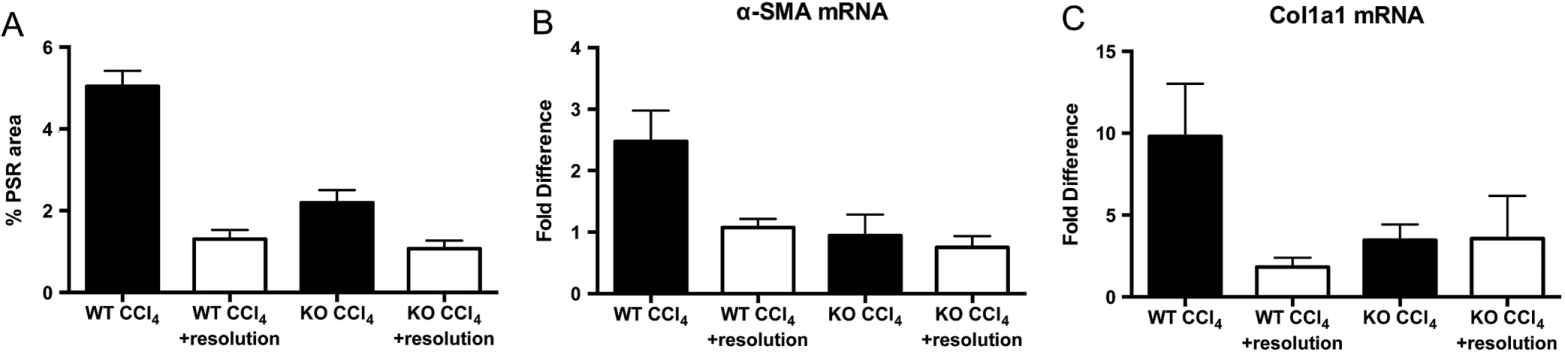
CD248 does not alter resolution of liver fibrosis. CD248^−/−^ mice and wild-type (WT) mice were injected biweekly with carbon tetrachloride (CCl_4_) or vehicle for 8 weeks and then sacrificed either 2 days or after 4 weeks of the final dose (resolution) (n=3–6). (A) The percentage fibrotic areas were assessed by ImageJ analysis of Picrosirius red stained (PSR) microphotographs taken of six randomly selected areas. (B) Col1a1 and (C) α-smooth muscle actin (α-SMA) gene expression analysis was performed with qPCR using total liver mRNA. Data are mean±SEM. *p<0.05, ***p<0.001 (Student's t test).

To establish whether resolution of fibrosis was altered in CD248^−/−^ mice, we treated WT and CD248^−/−^ mice for 8 weeks with CCl_4_ and examined their livers 4 weeks after the end of injury. After 4 weeks resolution, WT and CD248^−/−^ mice had similar levels of liver fibrosis (figure 7A). Levels of α-SMA and Col1a1 mRNA were also similar in the two groups (figure 7B, C).

### CD248-deficient mice are protected from hepatic fibrosis secondary to reduced HSC proliferation in response to PDGF-BB

**Figure 8:**
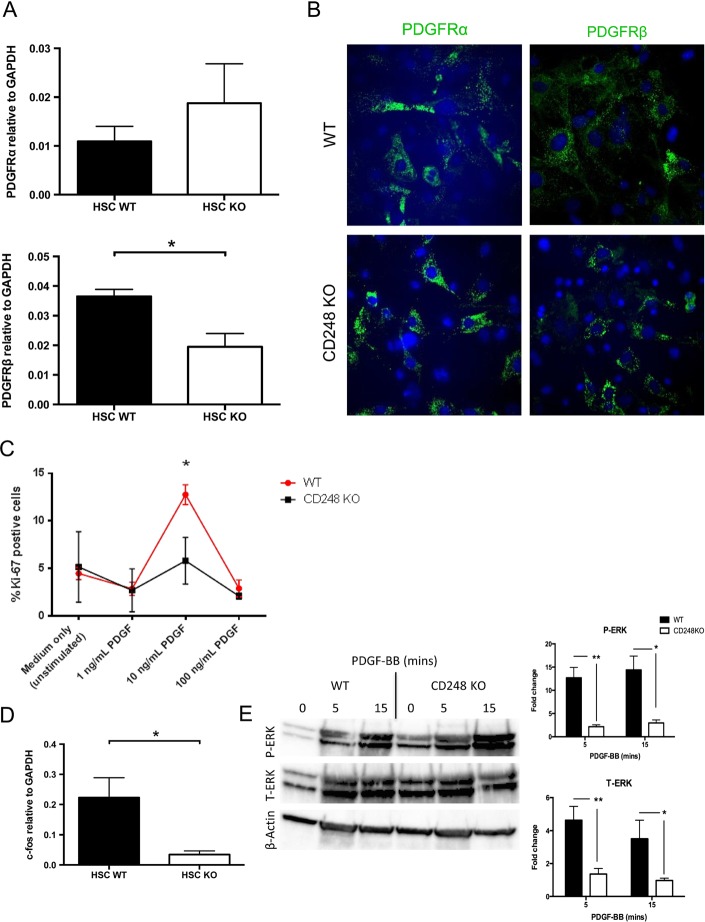
Platelet-derived growth factor (PDGF)-induced proliferation is impaired in hepatic stellate cells (HSCs) from CD248^−/−^ mice. HSCs were isolated from normal liver tissue from wild-type (WT) and CD248^−/−^ mice and plated on plastic to initiate activation. (A) Transcript levels of PDGF receptor (PDGFR)-α and PDGFR-β was analysed by qPCR (n=3). (B) Representative images of immunofluorescent staining of HSCs with antibodies against PDGFR-α and PDGFR-β are shown. (C) After stimulation with PDGF-BB, the number of WT and CD248^−/− HSCs^ (n=5) proliferating was numerated by the percentage that were Ki-67 positive. (D) qPCR was used to analyse c-fos expression in WT and CD248^−/−^ HSCs after stimulation with PDGF-BB (E) Protein analysis by western blot for expression of phosphorylated ERK (P-ERK) and total ERK (T-ERK) relative to β-actin in WT and CD248^−/−^ HSCs following treatment with 100 ng/mL PDGF-BB. A representative blot is shown with quantification by digital densitometry. Data are normalised to β-actin and expressed as fold change from unstimulated cells (n=2 independent experiments). Data are mean±SEM. *p<0.05, **p<0.01 (Student's t test). GAPDH, glyceraldehyde 3-phosphate dehydrogenase

To understand the mechanisms by which CD248 exerts its effects on fibrogenesis, we sought to establish whether PDGF, which is a known potent HSC mitogen and which is known to interact with CD248, played a role. Isolated HSCs from WT and CD248^−/−^ mice both expressed PDGFR-α mRNA and protein at similar levels (figure 8A, B). Although PDGFR-β mRNA was slightly lower in HSCs from CD248^−/−^ mice, expression levels of PDGFR-β protein did not show differences between HSCs from WT and CD248^−/−^ mice, indicating that they could respond to PDGF stimulation (figure 8A, B).

Although CD248^−/−^ HSC expressed PDGFR-α and PDGFR-β, they did not respond to stimulation with PDGF-BB in the same way as WT HSC. After exposure to PDGF-BB at a dose of 10 ng/mL, WT HSCs exhibited a marked proliferative response compared with HSCs from CD248^−/−^ mice (figure 8C). The downstream effects of abrogated PDGF signalling in CD248^−/−^ HSCs were confirmed by a demonstration of significantly reduced c-fos mRNA expression (0.03±0.01 vs 0.22±0.06, p<0.05) compared with that seen in HSCs from WT mice (figure 8D). Similarly after PDGF exposure, there was blunted phosphorylation of ERK by CD248^−/−^ HSCs compared with WT HSC. As we have reported previously, baseline levels of phospho-ERK were higher in CD248^−/−^ HSCs (figure 8E).

## Discussion

In this article, we demonstrate for the first time that CD248 is a major regulator of liver fibrosis in response to chronic injury, which is upregulated in liver fibrosis in both human and mouse settings. We demonstrate that genetic deletion of CD248 protects against the development of fibrosis in a murine model of chronic hepatocellular injury by interfering with PDGF signalling, and therefore represents a novel target for therapeutic intervention in fibrotic liver injury.

HSCs are considered to be the key cells that contribute to liver fibrosis.^13^ ^14^ The expression of CD248 in activated HSC and myofibroblasts prompted us to assess the role of CD248 in liver fibrogenesis. We showed that in an experimental model of liver fibrosis mice lacking CD248 developed less fibrosis, which may be secondary to a decrease in PDGF signal transduction resulting in defective HSC proliferation, and hence reduced ECM deposition. PDGF is the most potent HSC mitogen known and is a key regulator of HSC proliferation during hepatic fibrogenesis.^15^ A recent study by Tomkowicz *et al*^16^ has shown that CD248 expression is necessary for PDGF signalling in murine pericytes. Deficiency of CD248 in HSCs did not alter PDGF receptor levels, suggesting that the antiproliferative effect of CD248^−/−^ HSCs was not mediated through the modulation of PDGF receptor expression. Notably our studies indicate that PDGF signalling in CD248^−/−^ HSCs was abrogated as demonstrated by the significantly reduced *c-fos* expression following PDGF signalling in CD248^−/−^ HSC.

PDGF-BB binding to PDGF receptor causes PDGF receptor phosphorylation, which induces phosphorylation of ERK.^17^ The role of CD248 is in regulating this phosphorylation of ERK,^18^ and thus in the absence of CD248 ERK is not phosphorylated in response to PDGF. Phosphorylation of ERK leads to c-fos transcription, which then mediates the downstream effects (such as proliferation) that are used as a measure of PDGF signalling. Indeed, it has previously been suggested that modulating the PDGF signalling pathway could represent a strategy for guided tissue regeneration or tissue engineering of bone.^19^ In this study, we demonstrate that phosphorylation of ERK by PDGF was blunted by the absence of CD248, but not to the same extent as we reported with osteoblasts. Therefore, targeting of CD248 may allow selective inhibition of PDGF signalling only on activated HSCs and myofibroblasts in liver injury.

Despite a decreased proliferative potential of HSCs from CD248^−/−^ mice, there may be other mechanisms that are involved in reducing the levels of fibrosis in CD248^−/−^ mice. Other investigators have reported that CD248 may reduce the levels of inflammation. CD248^−/−^ mice had significantly less inflammation than their WT controls in an arthritis model with a decrease in leucocytes and proinflammatory cytokines.^20^ Notably we did not observe differences in inflammation or angiogenesis in CD248^−/−^ mice indicating a greater role in fibrogenesis.

The restricted expression of CD248 in HSCs and myofibroblasts and the temporal regulation during disease makes CD248 an attractive target for the modulation of liver fibrosis. Of note, resolution of fibrosis was unchanged in CD248^−/−^ mice. In addition, CD248 is expressed on the cell surface, and therefore CD248-positive stromal cells could be a potential target for the modulation of liver fibrosis using novel antiangiogenic drugs such as the antihuman CD248 monoclonal antibody MORAb-004,^21^ currently in clinical trials in cancer.

In this study, we have identified CD248 as a novel therapeutic target in liver fibrosis. Importantly, our study demonstrates that interfering with CD248 signalling significantly reduces hepatic fibrosis in a mouse model of chronic liver disease. Our data suggest that CD248 could be a new treatment target in fibrotic liver disease in humans.

## Supplementary Material

Web figure
