# Supplementary material for: CD248/endosialin critically regulates hepatic stellate cell proliferation during chronic liver injury via a PDGF-regulated mechanism
Source: Gut. 2015 Jun 15;65(7):1175–85. doi: 10.1136/gutjnl-2014-308325 (PMC4941145; doi:10.1136/gutjnl-2014-308325)

Supplementary Figure 1

CD45 and F4/80 staining was performed on control and CCl4 injured WT and CD248-/- mice to assess presence of pan-lymphocyte and macrophage populations.

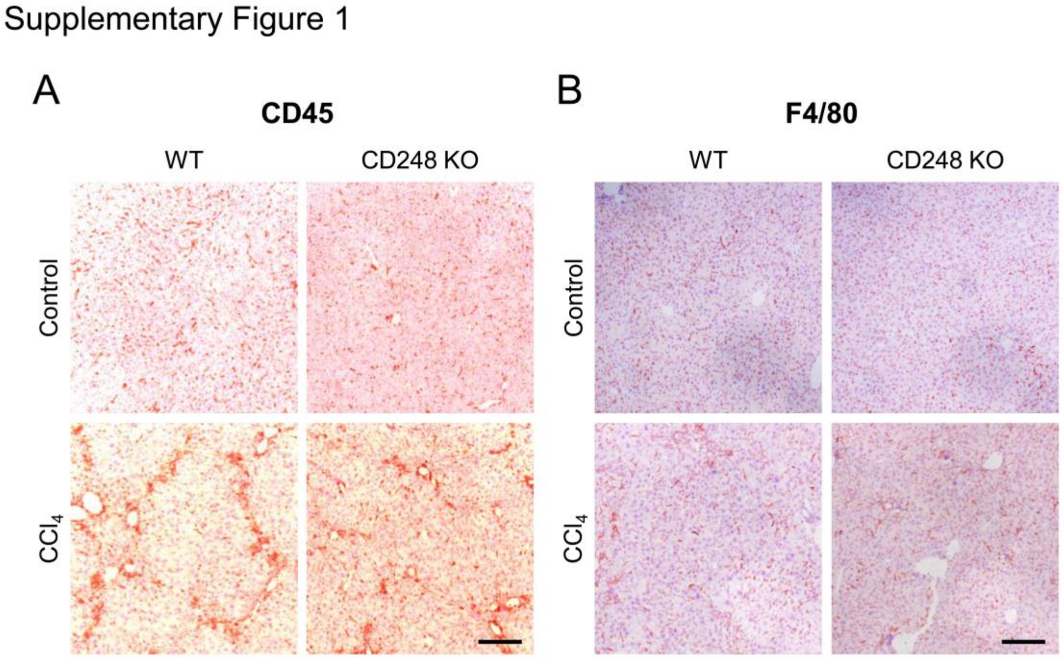

Supplement: Web figure [file gutjnl-2014-308325-s1.pdf]
